# HIV/AIDS and Forces

**DOI:** 10.4103/0970-0218.51216

**Published:** 2009-04

**Authors:** Neerja Jindal, Usha Arora

**Affiliations:** Governmet Medical College, Amritsar (Punjab), India. E-mail: neerjarajender@hotmail.com

Sir,

Acquired Immune Deficiency Syndrome (AIDS) is the most disastrous disease of its time for every segment of society. It is all the more dangerous for the forces (the people who are engaged in jobs where physical fitness is a prerequisite-Punjab police, Central police, Paramilitary forces and Army). Ever since the first HIV case in our armed forces was discovered in a soldier returning from a UN mission in 1992, the number of HIV-positive personnel in the armed forces has grown to 6,180 in a force of 12 lakhs.(1) The situation is the worst among the Central police and Para military forces.(1) In continuing with our effort to monitor the prevalence of HIV infection in these forces, we report the presence of infection in 712 personnel of all the forces (Punjab police 670; Central police and Para military forces 30; Army 12) who visited Integrated Counceling and Testing Centre (ICTC) of the Govt. Medical College during 2006. Of these 712 people, 660 (92.7%) were direct walk-ins and 52 (7.3%) were referred to ICTC with a suspicion of HIV infection because of the presence of HIV indicator diseases. We observed that during that year, 3.9% (22/712) of the personnel were HIV positive, which showed a gradual increase from HIV prevalence observed during 2005 (2.5%).(2) Although the difference between the rates observed in the 2 years was statistically insignificant (*P*>0.05), this is not of much importance. A comparison of the risk factors of the 2 years showed that during 2006, all the 22 seropositive cases had multiple sex partners and in two cases there was history of IVDU (Intravenous Drug Use) as well. No personnel were infected through contaminated blood or syringes/needles. This is in contrast to the findings in 2005 where positivity through the sexual route was 82.7% (6/7) and through blood transfusion was 17.3% (1/7). This shows an awareness about HIV/AIDS but very low use of safe sex practices 2.8% (20/712). A study conducted by experts under the National AIDS Control Organisation (NACO) reported that the Central police and Para military forces were not properly equipped to handle the serious problem of HIV/AIDS and interventions were ad hoc and uncoordinated. They also observed that the attitude towards HIV-positive personnel whose numbers were increasing every day was quite discriminating.(1) AIDS spreads its net mainly because it is treated as a stigma and such a mind set only leads to the proliferation of the problem. The Armed Forces need to accept HIV/AIDS and not hide it. This could be done by strengthening their organization and institutional structure and by sensitizing the personnel of the forces to all aspects of the problem.
